# Prevalence, distribution, and severity of cerebral amyloid angiopathy differ between Lewy body diseases and Alzheimer’s disease

**DOI:** 10.1186/s40478-023-01714-7

**Published:** 2024-02-15

**Authors:** Lauren Walker, Harry Simpson, Alan J. Thomas, Johannes Attems

**Affiliations:** Translational and Clinical Research Institute, Newcastle University, Edwardson building, Campus for Ageing and Vitality, Newcastle-upon-Tyne, NE4 5PL UK

**Keywords:** Cerebral amyloid angiopathy, Dementia with Lewy bodies, Parkinson’s disease dementia, Parkinson’s disease, Alzheimer's disease

## Abstract

Dementia with Lewy bodies (DLB), Parkinson’s disease dementia (PDD), and Parkinson’s disease (PD) collectively known as Lewy body diseases (LBDs) are neuropathologically characterised by α-synuclein deposits (Lewy bodies and Lewy neurites). However, LBDs also exhibit pathology associated with Alzheimer’s disease (AD) (i.e. hyperphosphorylated tau and amyloid β (Aβ). Aβ can be deposited in the walls of blood vessels in the brains of individuals with AD, termed cerebral amyloid angiopathy (CAA). The aim of this study was to investigate the type and distribution of CAA in DLB, PDD, and PD and determine if this differs from AD. CAA type, severity, and topographical distribution was assessed in 94 AD, 30 DLB, 17 PDD, and 11 PD cases, and *APOE* genotype evaluated in a subset of cases where available. 96.3% AD cases, 70% DLB cases and 82.4% PDD cases exhibited CAA (type 1 or type 2). However only 45.5% PD cases had CAA. Type 1 CAA accounted for 37.2% of AD cases, 10% of DLB cases, and 5.9% of PDD cases, and was not observed in PD cases. There was a hierarchical topographical distribution in regions affected by CAA where AD and DLB displayed the same distribution pattern that differed from PDD and PD. *APOE* ε4 was associated with severity of CAA in AD cases. Topographical patterns and severity of CAA in DLB more closely resembled AD rather than PDD, and as type 1 CAA is associated with clinical dementia in AD, further investigations are warranted into whether the increased presence of type 1 CAA in DLB compared to PDD are related to the onset of cognitive symptoms and is a distinguishing factor between LBDs. Possible alignment of the the topographical distribution of CAA and microbleeds in DLB warrants further investigation. CAA in DLB more closely resembles AD rather than PDD or PD, and should be taken into consideration when stratifying patients for clinical trials or designing disease modifying therapies.

**Supplementary Information:**

The online version contains supplementary material available at 10.1186/s40478-023-01714-7.

## Introduction

Neurodegenerative diseases of the ageing brain are defined neuropathologically by their most prevalent pathology, using internationally recognised staging criteria. However, it is rare that pathologies exist in isolation, with pure pathology only seen in 22.7% of *post-mortem* cases in a large neuropathological study consisting of 670 brains [40]. Although low/intermediate or indeed severe/high levels of concomitant pathology are present across numerous diseases, they are particularly evident across Lewy body diseases (LBDs) including dementia with Lewy bodies, Parkinson’s disease (PD), and Parkinson’s disease dementia (PDD), where in addition to hallmark inclusions of α-synuclein, concomitant AD related pathology (intracellular tau-immunoreactive neurofibrillary tangles and extracellular amyloid β (Aβ) plaques) is a prominent feature [17, 35, 39, 61, 62].

Cerebral amyloid angiopathy (CAA), observed in 20–100% of AD cases [20, 32, 38, 48], is defined by the deposition of Aβ (predominantly Aβ_1-40_) in the walls of meningeal vessels, cerebral arteries, arterioles, and less commonly in the capillaries and vein vessel walls [28, 49, 67]. It exists in two forms, the first (type 1 CAA) affects the capillaries, with or with-out involvement of cortical or leptomeningeal vessels, the second (type 2 CAA) includes Aβ deposition restricted to leptomeningeal and cortical arteries, arterioles, and rarely veins without capillary involvement [54].

The association of CAA with clinical dementia has been investigated, 87.5% of individuals with dementia have CAA, whilst only 55.6% of cognitively normally individuals exhibit CAA at *post-mortem* examination [4]. CAA has also been identified as a contributor to cognitive decline independent of other AD related neuropathology [7, 56]. Furthermore, type 1 CAA was found to mildly correlate with clinical dementia and AD neuropathology, whereas type 2 CAA did not [4].

Although a frequent observation in AD at *post-mortem* examination, CAA has also been reported in LBDs with recent studies reporting between 91 and 100% of DLB cases, 50–63% of PDD cases, and 13% of PD cases displaying CAA pathology [25, 29]. The increased presence of type 1 CAA has also been reported in LBD cases with dementia as demonstrated by a series of 88 cases including PD, PDD and DLB cases. Whilst only 25% of PD cases exhibited type 1 CAA, DLB and PDD cases exhibited higher rates of type 1 CAA deposition (90% and 85% of cases respectively) [30].

Studies suggest that neurodegenerative pathologies spread in a predictable stereotypical manner i.e. tau pathology originates in the entorhinal cortex and progresses to the neocortex [1, 8] and Aβ starts in the neocortex and advances through the limbic system and brainstem to the cerebellum [55]. α-synuclein originates in the brainstem, progresses through the limbic regions and to the neocortex in PD/PDD [9, 42], with a limbic -predominant profile being more associated with cognitive decline in DLB [58]. However, in the presence of multiple pathologies typical topographical patterns of distribution may be altered. Lewy related pathology in DLB cases with significant concomitant AD related pathology shows a different distribution to DLB cases with minimal AD related pathology, and both groups differ from PD cases [58, 61]. With respect to CAA in AD, the occipital cortex is the most commonly and severely affected brain region in AD, with frontal, parietal, and temporal lobes less affected [3, 59, 64]. However, distribution patterns of CAA have yet to be investigated in LBDs, and as DLB cases can exhibit a greater Aβ burden compared to PDD and PD cases [18, 24, 31] whether LBDs have a similar distribution of CAA is currently unknown.

Therefore, the aim of this study was to investigate differences in type, topographical distribution of CAA, and association with dementia, across the spectrum of LBDs and determine if this differs from AD cases.

## Materials and methods

### Study cohort

Brain tissue from 152 donors (mean age 81.39 ± 9.6 years; male: 89, female: 63) was used in this study. 94 cases fulfilled neuropathological criteria for high AD neuropathologic change according to NIA‐AA criteria [45] inclusive of Braak stage [8], Thal phase [55] and CERAD score [44], and clinically diagnosed with AD [43]. 47 cases had limbic/neocortical Lewy body disease [42] (30 clinically diagnosed as dementia with Lewy bodies (DLB) and 17 with Parkinson’s disease dementia), and 11 diagnosed with Parkinson’s disease [42]. Cases that fulfilled neuropathological criteria for mixed AD/DLB (i.e. DLB with high AD neuropathologic change) were excluded from the study. Patient demographics including neuropathological characteristics are shown in Table 1. There was no significant difference in age at death between any of the groups (*p* = 0.067). There was no difference between sex in the AD group (*p* = 0.83) or PDD group (*p* = 0.71), however there was significantly more males than females in the DLB and PD groups (DLB *p* = 0.003; PD *p* = 0.035). Clinical records were systematically reviewed by a board-certified Old Age Psychiatrist (AJT). Brain tissue was obtained at autopsy and stored within the Newcastle Brain Tissue Resource (NBTR) in accordance with Newcastle University Ethics Board (The Joint Ethics Committee of Newcastle and North Tyneside Health Authority, reference: 08/H0906/136). After autopsy the right hemisphere, brainstem and cerebellum were immersion fixed in 4% buffered aqueous formaldehyde solution for 6 weeks. Irrespective of clinical diagnoses, all brains underwent neuropathological assessment and were stratified by clinico-pathological consensus. *APOE* genotype was provided by the Newcastle Brain Tissue Resource.

**Table 1:** Demographics of cohort

	AD	DLB	PDD	PD	p value^‡^
case n	94	30	17	11	
Age (± SE)	82.8 (± 0.91)	79.4 (± 0.91)	82.7 (± 1.63)	79.1 (± 2.94)	0.067
Sex (M:F)	45:49	23:7	12:5	9:2	p < 0.05^ab§^
Braak NFT stage [8]	Braak IV = 1 Braak V = 5 Braak VI = 88	Braak I = 3 Braak II = 8 Braak III = 15 Braak IV = 4	Braak I = 2 Braak II = 2 Braak III = 11 Braak IV = 2	Braak I = 4 Braak II = 4 Braak III = 3	p < 0.001^c§^
CERAD score [44]	B = 10 C = 84	negative = 17 A = 4 B = 7 C = 2	negative = 10 A = 4 B = 3	negative = 11	p < 0.001^de§^
Thal Phase [55]	Thal 4 = 6 Thal 5 = 88	Thal 0 = 2 Thal 1 = 2 Thal 2 = 3 Thal 3 = 8 Thal 4 = 8 Thal 5 = 7	Thal 0 = 1 Thal 1 = 2 Thal 2 = 3 Thal 3 = 4 Thal 4 = 3 Thal 5 = 4	Thal 0 = 4 Thal 1 = 2 Thal 2 = 2 Thal 3 = 2 Thal 4 = 1	p < 0.001^f§^
NIA-AA criteria [45]	Intermediate = 1 High = 93	Not = 2 Low = 15 Intermediate = 13	Not = 1 Low = 11 Intermediate = 5	Not = 4 Low = 6 Intermediate = 1	p < 0.001^gh§^
McKeith criteria [42]	no LBD = 69 Brainstem = 2 Amygdala predominant = 23	Limbic = 4 Neocortical = 28	Limbic = 1 Neocortical = 16	Brainstem = 8 Limbic = 3	p < 0.001^ij§^
Braak LB stage [9]	Braak 0 = 69 Braak 1 = 2 not classifiable = 23	Braak 4 = 4 Braak 5 = 1 Braak 6 = 25	Braak 4 = 1 Braak 5 = 3 Braak 6 = 13	Braak 2 = 3 Braak 3 = 5 Braak 4 = 3	p < 0.001^klm§^

^a^In DLB M > F, *p* < 0.01

^b^In PDD M > F, *p* < 0.05

^c^AD > DLB, PDD, and PDD all *p* < 0.001

^d^AD > DLB, PDD, and PD all *p* < 0.001

^e^PDD > PD, *p* < 0.05

^f^AD > DLB, PDD, and PD all *p* < 0.001

^g^AD > DLB, PDD, and PD all *p* < 0.001

^h^DLB > PD, *p* < 0.05

^i^AD vs DLB, PDD, and PD all *p* < 0.001

^j^DLB and PDD vs PD both *p* < 0.001

^k^AD vs DLB, PDD, and PD all *p* < 0.001

^l^DLB vs PDD and PD both at least *p* < 0.05

^m^PDD vs PD *p* < 0.001

^‡^chi-square test

^§^Fisherss exact test

### Immunohistochemistry

Paraffin embedded blocks taken from the frontal (Brodmann area (BA) 9), temporal (BA 21/22), parietal (BA 40), and occipital (BA17/18) cortices were cut at 6 μm and airdried onto superfrost plus charged glass slides (Thermo Shandon, Cheshire, UK). Tissue sections then underwent immunohistochemical staining for Aβ (4G8, dilution 1:15,000, Signet Labs, Dedham, MA, USA) for the detection of Aβ deposits in vessels (CAA) and extracellular Aβ plaques. Prior to immunostaining antigen retrieval was performed by immersing slides for 1 h in 100% formic acid. Immunopositivity was detected using a MENAPATH HRP polymer detection kit (Menarini diagnostics, Berkshire, UK) with 3,3 diaminobezidine (DAB) as a chromagen and haematoxylin as counter stain. Tissue was subsequently dehydrated through a series of alcohols, cleared and mounted using DPX (CellPath, Powys, UK).

### Neuropathological scoring

**Fig. 1:**
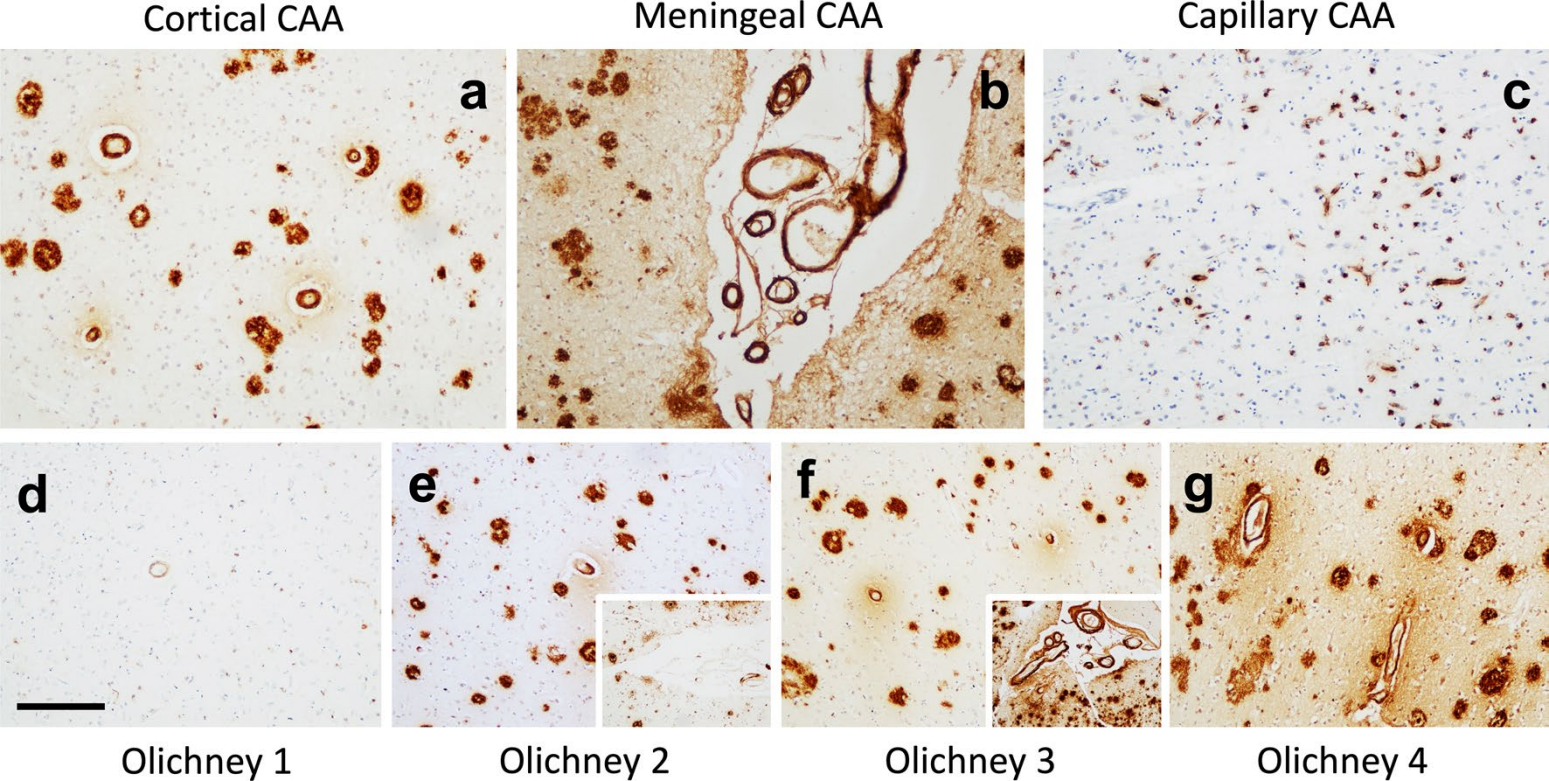
Photo micrographs demonstrating the types and semi-quantitative scoring of cerebral amyloid angiopathy (CAA). **a** Cortical CAA, **b** leptomeningeal CAA, and **c** capillary CAA. For this study the Olichney scoring system (41) was used and comprises of 4 grades **d** Olichney 1 A trace to scattered positivity in leptomeningeal or cortical vessels, **e** Olichney 2 —some cortical or leptomeningeal vessels circumferentially affected, **f** Olichney 3 A widespread circumferential CAA, and **g** Olichney 4— widespread circumferential CAA, with dyshoric changes surrounding the vessel. Scale bar represents 200 μm

Each case (inclusive of frontal, temporal, parietal, and occipital lobes) was assessed for type and severity of CAA according to standardised neuropathological staging criteria described previously by Thal [54], Olichney [47] and the consensus prototcol for the assessment of CAA [36]. The agreed protocol in the consensus scores parenchymal and meningeal CAA on a 0–4 scale and capillary CAA as present/absent in the 4 cortical lobes. The semi-quantitative scoring for parenchymal and meningeal CAA was recorded as follows: 0, no Aβ present in vessel walls; 1, scant Aβ deposition; 2, some circumferential Aβ deposits; 3, widespread, circumferential Aβ positivity; 4, as for 3 with additional dyshoric changes (Fig. 1). Inter-rater reliability for CAA is high [36]. However, we are not aware of studies on the intra-rater reliability for Olichney scoring, but there will probably be some degree of intra-rater inconsistency similar to the majority of other semi-quantitative scoring methods. To calculate the overall cortical CAA score, semi-quantitative scores for each brain region (frontal, temporal, parietal, and occipital) were combined [5].

### Statistical analysis

The Statistical Package for Social Sciences software (SPSS ver. 26) was used for statistical evaluation. Variables were tested for normality using the Shapiro-Wilk test and visual inspection of variable histograms. Group effects were assessed using either non-parametric (Mann-Whitney U) or parametric (independent samples t-test) procedures. Chi-squared tests or Fisher’s exact test were used to assess differences in categorical variables. A *p* value of < 0.05 was considered significant.

## Results

### Presence of CAA and differences in CAA type

**Fig. 2:**
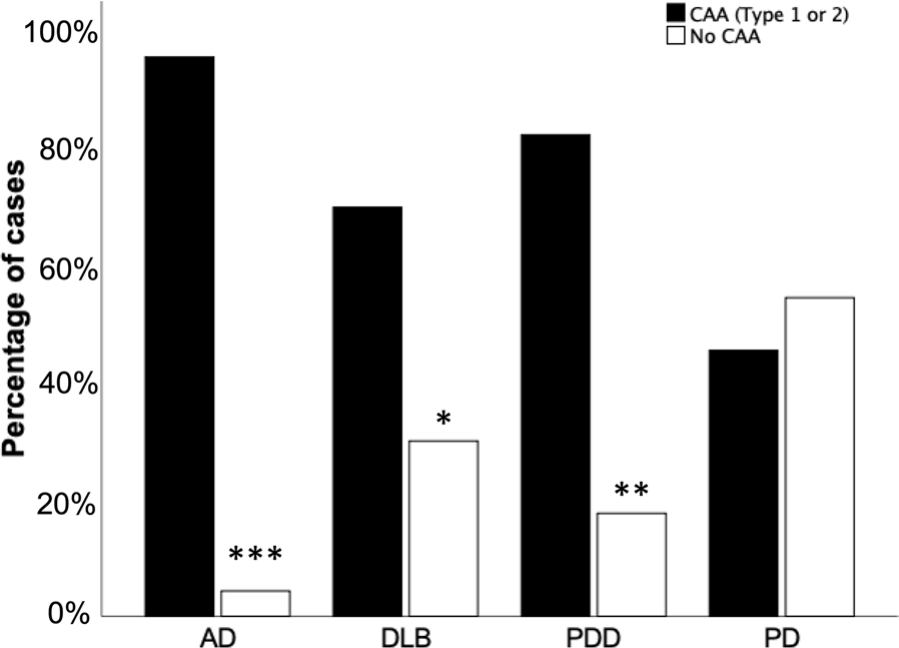
Significantly more AD, DLB, PDD and PD cases had CAA (type 1 or type 2) compared to to those without. There were no differences in the number of PD cases with and without CAA. *p < 0.05 **p < 0.01 ***p < 0.001. Chi-square test used. Abbreviations: AD; Alzheimer’s disease, DLB; dementia with Lewy bodies, PDD; Parkinson’s disease dementia, PD; Parkinson’s disease

**Fig. 3:**
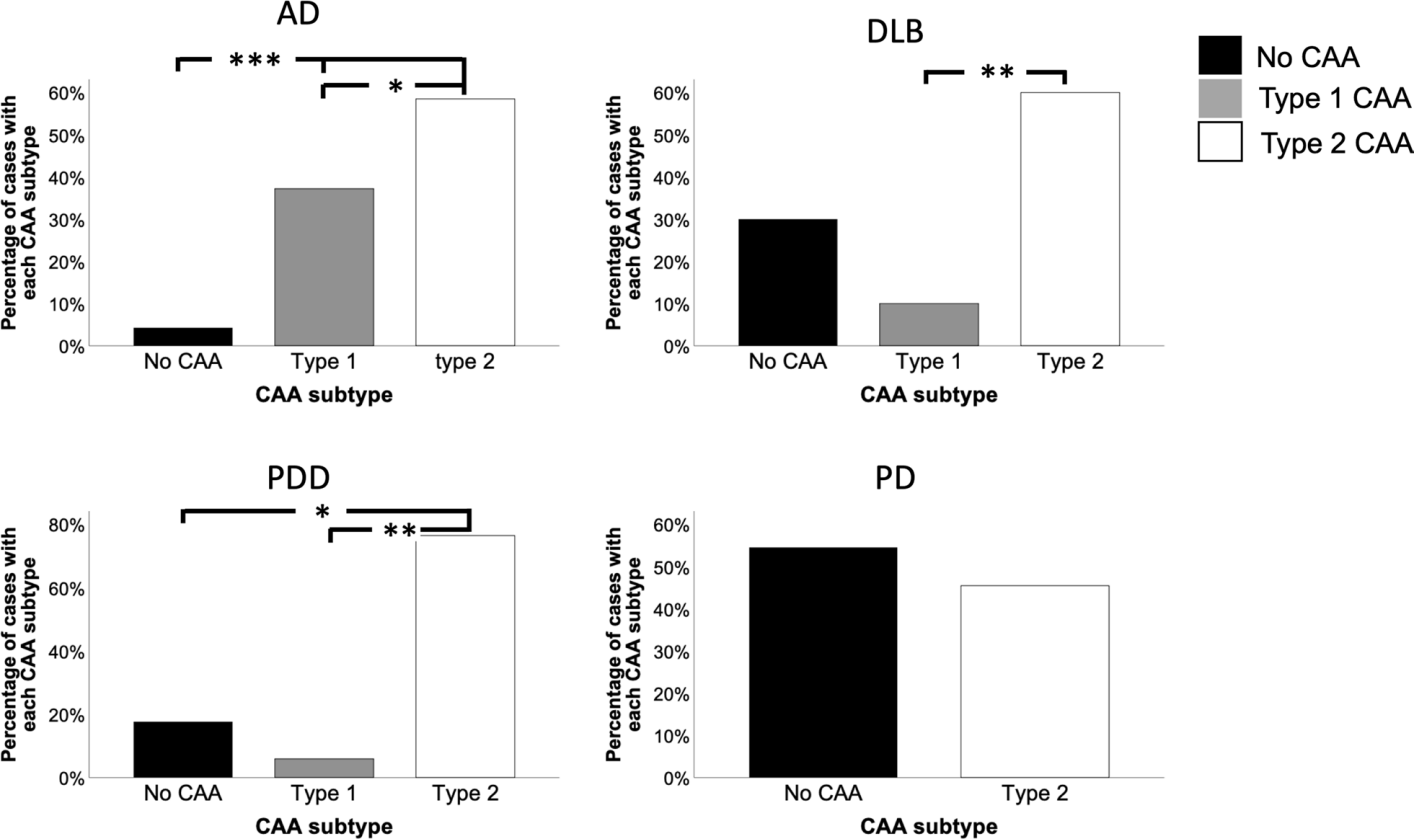
Prevalence of CAA subtype across each disease group. Abbreviations: AD, Alzheimer’s disease; DLB, dementia with Lewy bodies; PDD, Parkinson’s disease dementia; PD, Parkinson’s disease. Black bars represent cases with no CAA, gray bars represent cases with type 1 CAA, and white bars represent cases with type 2 CAA. *p < 0.05 **p < 0.01 ***p < 0.001 using chi-square test

96.3% AD cases, 70% DLB cases and 82.4% PDD cases exhibited CAA (Type 1 or Type 2). However only 45.5% PD cases had any type of CAA. There was no difference in the number of PD cases that had CAA compared to those without CAA (*p* > 0.05) (Fig. 2). The most frequent type of CAA across all disease groups was type 2 CAA, with AD (*p* < 0.05), DLB and PDD (both *p* < 0.01) exhibiting significantly more type 2 CAA compared to type 1 CAA. Those neuropathologically categorised as type 1 CAA accounted for 37.2% of AD cases, 10% of DLB cases, and 5.9% of PDD cases. Within our cohort, type 1 CAA was not observed in any PD cases (Fig. 3).

### Severity of CAA scores across disease groups

**Fig. 4:**
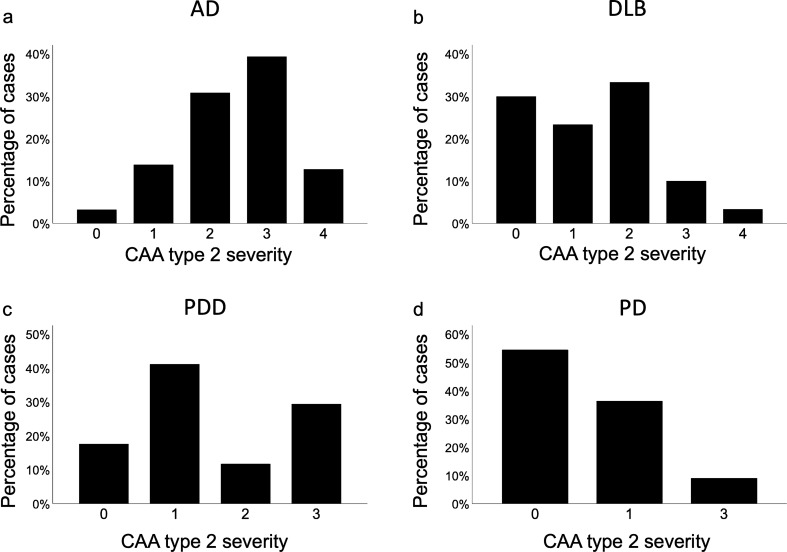
Severity CAA scores in cases classified with type 2 CAA across disease categories. Both AD (**a**) and DLB (**b**) groups contained cases that reached the highest grading of CAA (Olichney 4), however this most severe score was not given to any case in the PDD (**c**) or PD (**d**) groups. Abbreviations: AD; Alzheimer’s disease, DLB; dementia with Lewy bodies, PDD; Parkinson’s disease dementia, PD; Parkinson’s disease

**Fig. 5:**
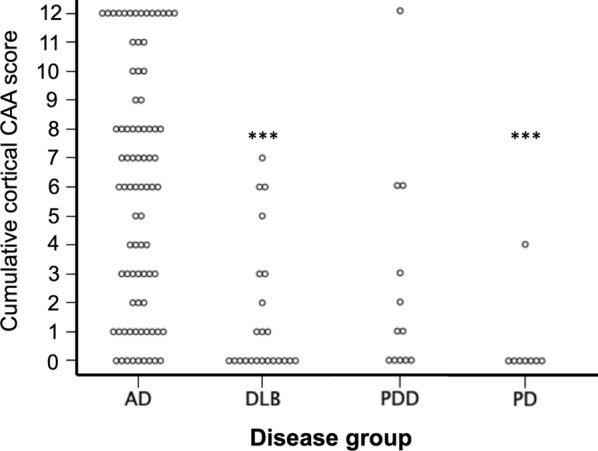
Overall mean cumulative cortical CAA scores across different groups. AD had a significantly higher mean cumulative CAA score compared to DLB (***p < 0.001), PDD (**p < 0.01), and PDD (***p < 0.001) using chi-square tests. There were no differences between any other groups

Further analysis investigated the severity of CAA in cortical and meningeal vessles, (this was not available for capillary CAA as criteria only stipulate presence or absence). Cases in both AD and DLB groups exhibited the highest CAA score Olichney 4 (widespread, circumferential Aβ positivity in both leptomeningeal and intercortical vessels with dyshoric changes), whilst no cases in the PDD or PD groups exceeded an Olichney score of 3 (Fig. 4). When assessing the overall cortical CAA score, AD was significantly higher than DLB (*p* < 0.001) and PD (*p* < 0.001). There were no differences between any other groups (Fig. 5). When looking at severity scores for cortical and meningeal CAA across individual brain regions AD cases had significantly increased CAA scores compared to DLB, PDD, and PD in all regions assessed (for breakdown of individual regions see Table 2). With respect to differences between the LBD groups, meningeal CAA was significantly increased in DLB compared to PD in the frontal cortex (*p* < 0.05), whilst in the occipital cortex both meningeal and cortical CAA was increased in PDD compared to PD (both *p* < 0.05).

**Table 2:** Severity scores for cortical and meningeal CAA across individual brain regions

		AD Median (range)	DLB Median (range)	PD Median (range)	PDD Median (range)	*p* value ^§^	
Frontal	Cortical	2 (0–3)	0 (0–3)	0 (0)	0 (0–3)	*p* < 0.001 ^a§^	
	Meningeal	2 (0–3)	1 (0–3)	0 (0–1)	0 (0–3)	*p* < 0.001^b§^	
Temporal	Cortical	0 (0–3)	0 (0–3)	0 (0–1)	0 (0–3)	*p* < 0.05^c§^	
	Meningeal	2 (0–3)	0 (0–3)	0 (0–3)	0 (0–3)	*p* < 0.001 ^d§^	
Parietal	Cortical	1 (0–3)	0 (0–3)	0 (0–1)	0 (0–3)	p < 0.01 ^e§^	
	Meningeal	2 (0–3)	0 (0–3)	0 (0–3)	0 (0–3)	*p* < 0.001 ^f§^	
Occipital	Cortical	2 (0–3)	0 (0–3)	0 (0–2)	1 (0–3)	*p* < 0.001 ^gh§^	
	Meningeal	3 (0–3)	1 (0–3)	0 (0–3)	2 (0–3)	*p* < 0.001 ^ij§^	

AD cases had significantly increased man CAA scores compared to DLB, PDD, and PD in all regions assessed. With respect to differences between the LBD groups, meningeal and cortical CAA was significantly different in PDD compared to PD in the occipital cortex (*p* < 0.05)

*AD* Alzheimer’s disease, *DLB* dementia with Lewy bodies, *PDD* Parkinson’s disease dementia, *PD* Parkinson’s disease

^a^AD > DLB, PD, and PDD all at least *p* < 0.05

^b^AD > DLB, PD, and PDD all *p* < 0.001

^c^AD > DLB *p* < 0.05

^d^AD > DLB, PD, all at least *p* < 0.001

^e^AD > DLB, PD, and PDD *p* < 0.05

^f^AD > DLB, PD, and PDD *p* < 0.05

^g^AD > DLB, PD, all at least *p* < 0.001

^h^PDD > PD *p* < 0.05

^i^AD > DLB, PD, all at least *p* < 0.001

^j^PDD > PD *p* < 0.05

^§^Fisher’s exact test

### Distribution patterns of CAA between brain regions

**Fig. 6:**
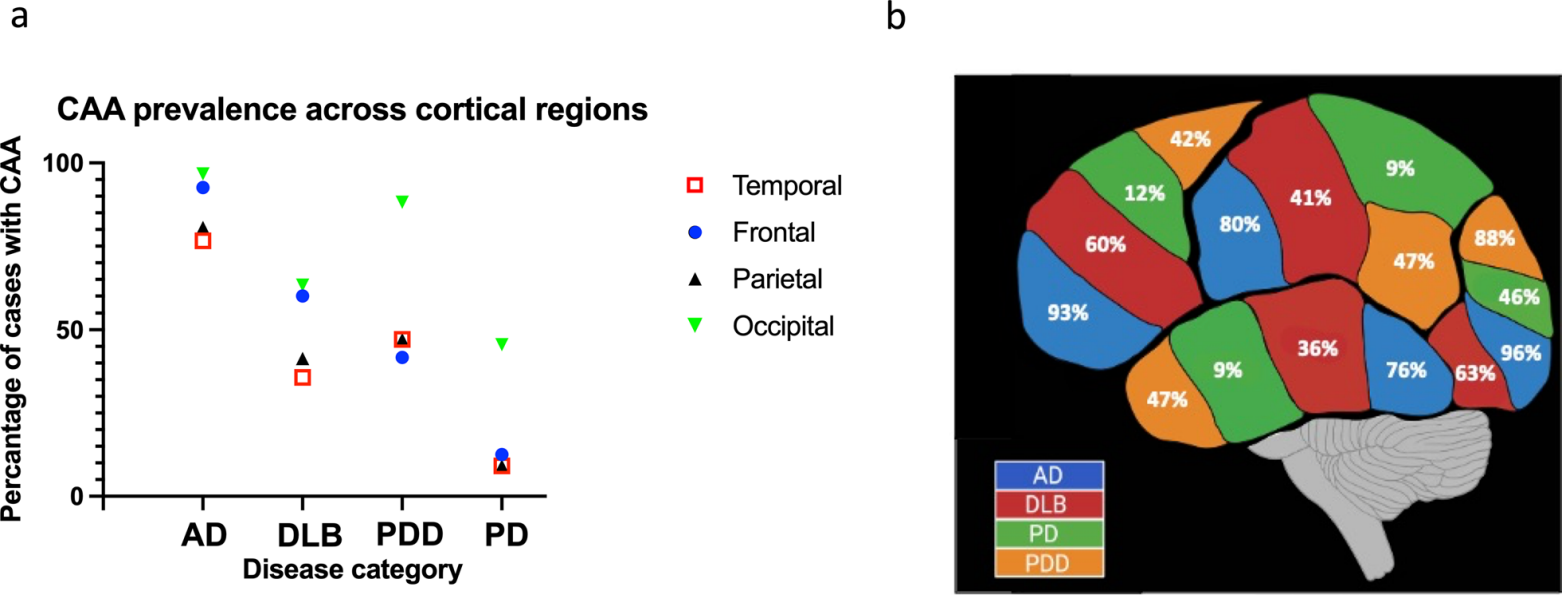
**a** Scatter graph demonstrating differences in topographical distribution across the difference disease groups. Occipital lobe was affected most across all diseases, with AD and DLB groups following the same hierarchical order occipital > frontal > parietal > temporal. This differed from PDD and PD groups where frontal cortex was one of the least affected brain regions. **b** visual representation of graphical data

The most commonly affected brain region across all diseases was the occipital lobe. Interestingly, the pattern of distribution was similar between AD and DLB cases with the frontal cortex being the next most commonly affected followed by the parietal and lastly the temporal cortex. This differs from the distribution pattern of CAA in PDD and PD where although the occipital cortex is the most commonly affected region the frontal cortex is the one of the least affected regions (Fig. 6).

### APOE genotype

**Fig. 7:**
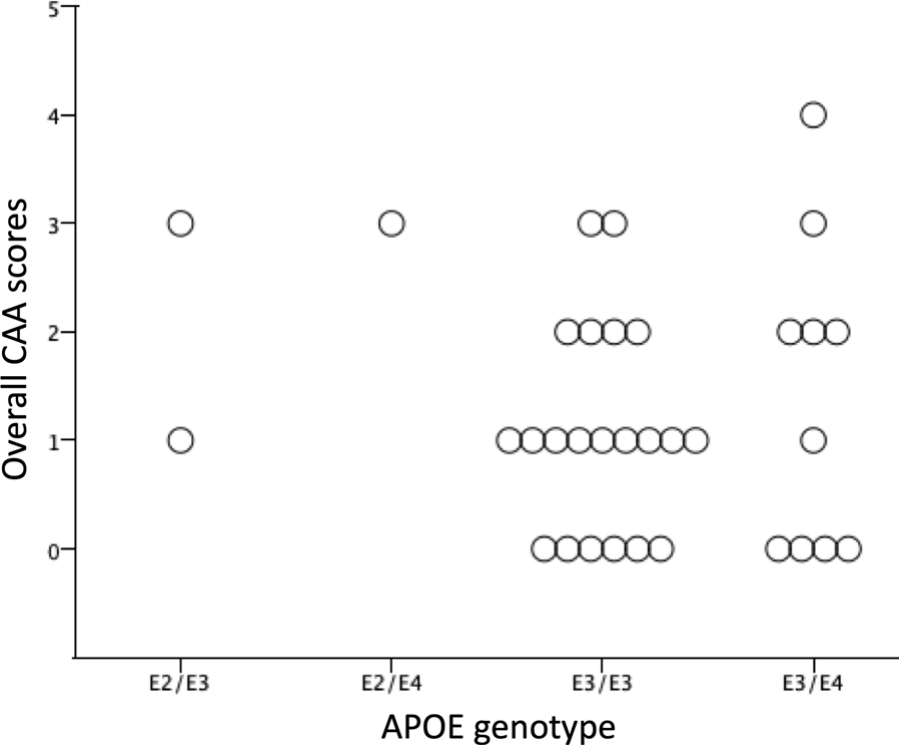
In LBD cohort (DLB, PDD, and PD combined) the genotypes with the highest mean Olichney scores were E2/E3 and E2/E4

*APOE* status was available in 96 cases (62 AD, 20 DLB, 10 PDD, and 4 PD). The most frequent genotype in AD was ε3/4 (59.7%), however in LBDs the most frequent genotype was ε3/3 (DLB 60%, PDD 70%, and PD 50%) Table 3, and CAA severity scores for each genotype is presented in Fig. 7. In the whole cohort the presence of an *APOE* ε4 allele was associated with CAA severity (*p* < 0.05), however no association was observed between CAA severity and the presence an *APOE* ε2 or *APOE* ε3 alleles. When considering individual disease groups the association between CAA severity and the presence of an *APOE* ε4 allele, this only remained significant in the AD group (*p* < 0.05). The presence of the ε4 allele was also associated with the presence of type 1 CAA (*p* < 0.05) in the overall cohort. No other associations were observed between *APOE* status and type 1 CAA.

**Table 3:** *APOE* genotype across all disease groups

*APOE* genotype	Overall N, (%)	AD N, (%)	DLB N, (%)	PDD N, (%)	PD N, (%)
	Total n = 96	Total n = 62	Total n = 20	Total n = 10	Total n = 4
e2/2	1 (1.04)	1 (1.51)	(0.00)	(0.00)	0.00
e2/3	3 (3.13)	1 (1.51)	1 (5.00)	(0.00)	1 (25.00)
e2/4	3 (3.13)	2 (3.32)	(0.00)	1 (10.00)	0.00
e3/3	35 (36.50)	14 (22.60)	12 (60.00)	7 (70.00)	2 (50.00)
e3/4	47 (48.95)	37 (59.70)	7 (35.00)	2 (20.00)	1 (25.00)
e4/4	7 (7.29)	7 (11.36)	(0.00)	(0.00)	0.00
*APOE* allele presence					
e2	(7.30)	(6.50)	(5.00)	(10.00)	(25.00)
e3	(88.50)	(83.90)	(100.00)	(90.00)	(100.00)
e4	(60)*†	(75.4)*†¶	(35.00)	(30.00)	(25.00)

*AD* Alzheimer’s disease, *DLB* dementia with Lewy bodies, *PDD* Parkinson’s disease dementia, *PD* Parkinson’s disease

^*^p < 0.05 using chi-square test

^*†^e4 is associated with CAA severity in whole cohort and AD

^¶^e4 is associated with presence of type 1 CAA in AD

### Effects of severe CAA on brain parenchyma

We next investigated if ischemic and haemorrhagic lesions were present in AD and DLB cases with severe CAA. Vascular pathology was present in 13/49 AD cases and 0/4 DLB cases. Both infarcts and microhaemorrhages were present in the AD cases with severe CAA (Additional file 1: Table S1, online resource).

## Discussion

Mixed pathologies, in particular AD related pathology is a frequent finding in LBDs, and data from this study adds to growing evidence to suggest CAA may be a contributing pathological substrate in LBDs, in particular in DLB. Building on previous studies that have demonstrated differences in the prevalence of CAA in DLB compared to PDD [25, 29, 30], here we report differences in the severity and the topographical distribution of CAA across the LBD spectrum.

In our study we found CAA is more common in cases with clinical dementia, as significantly more AD, DLB, and PDD cases had CAA, and there was no difference in the number of PD cases with and without CAA. This is in agreement with other studies, as evidence from a longitudinal study using data from 1100 well characterised older adults suggests that CAA is associated with faster rates of decline in global cognition, perceptual speed, and episodic and semantic memory over a 19 year period [7]. Furthermore, both large population and community-based studies have reported individuals with moderate to severe CAA perform worse on cognitive tasks [2, 10]. The disease groups with the highest CAA scores (Olichney grade 4) in our study are AD and DLB, with none of the PD or PDD groups (which originally start out as motor disorders, and potentially progressing to dementia) exhibiting the highest CAA score available. When considering the LBD cases only this finding is in alignment with a study by Jellinger where only DLB cases exhibited the highest CAA score, with neither PD or PDD cases displaying the highest severity level [29]. In the current study the cumulative cortical CAA scores were significantly higher in AD compared to DLB and PD, and there was no difference observed between AD and PDD. However, in the PDD group there was one case that exhibited severe CAA in all cortical regions which could be driving this. Type 1 CAA is more common in DLB cases compared to PDD, which is interesting given the association between type 1 CAA and cognitive decline [7, 56], and not present at all in our PD cohort. The relationship between type 1 CAA, AD related pathology and dementia has been reported in a population-based study using the Vantaa 85 + cohort, where the authors found type 1 CAA was associated with severity of generalised CAA and the presence of dementia [37]. Furthermore, in agreement with a previous neuropathological study DLB and PDD cases are more likely to have type 1 CAA compared to PD without dementia [30]. Although it is difficult to neuropathologically distinguish DLB from PDD, DLB cases have been shown to exhibit increased AD related pathology compared to PDD cases [31, 62]. Therefore to investigate if the differences in CAA severity between DLB and PDD are evident in the absence of abundant amyloid plaques in the parenchyma, this study was designed to exclude all LBD cases with high AD neuropathologic change. There were no differences in Thal phase, Braak neurofibrillary tangle stage or CERAD score between DLB and PDD groups, which is also in agreement with a study by Hansen and colleagues. They found no difference in Thal phases between DLB and PDD groups, however CAA in DLB was more severe compared to PDD [25]. The relationship between parenchymal amyloid load and CAA severity has been investigated in AD [57]. No association was observed between amyloid plaque load and CAA severity in individual brain regions, and it is speculated that differing pathogenic mechanisms underpin the deposition of amyloid in plaques and blood vessels. This is not surprising considering the predominant amyloid species in plaques are mostly peptides that are 42 amino acids in length whilst amyloid deposits in CAA mainly comprise of shorter peptides of 40 amino acids.

In the current study the topographical pattern of distribution of generalised CAA in DLB is similar to AD, and this differs from the distribution pattern observed in both PD and PDD. The most commonly affected brain region across all diseases was the occipital cortex. We did not look at differences in specific Brodmann areas in the occipital cortex i.e. Brodmann areas 17 and 18, as unlike tau pathology in Braak neurofibrillary tangle staging, where this delineates Braak stages V/VI, there is no protocol for this when assessing amyloid pathology. In AD and DLB the next most commonly affected brain region is frontal cortex whilst this is the least affected brain region in PD and PDD cases. Interestingly other reports have described concomitant pathology altering the topographical distribution of pathological protein aggregates in patients with multiple pathologies. Toledo and colleagues describe different clusters patterns of pathology in patients with clinical dementia and AD and Lewy related pathology compared to PD patients without AD related pathology [58], whilst a study from our group demonstrated the spread of hyperphosphorylated tau pathology in cases with mixed AD/DLB differs to that in ‘pure ADs [61]. It has been suggested that multiple pathologies promote suitable conditions for a synergistic relationship between proteins that results in cross seeding and exacerbation of overall pathology burden and accelerating cognitive decline [6, 13, 14, 21, 27]. To our knowledge this is the first study demonstrating divergence of pathology patterns in cerebral amyloid deposition in LBDs, and could, in part, be a product of increased synergistic relationships between cortical pathologies in DLB. From a clinical perspective the finding of significant involvement of CAA in the frontal cortex of patients with AD and DLB (predominantly cognitive neurodegenerative diseases) compared to patients with PD or PD (primarily diagnosed as motor disorders) is interesting. Two previous studies have suggested the presence of CAA is associated with impairments to executive function which is controlled by the frontal cortex [12, 63]. This raises questions regarding the potential contribution of CAA to specific cognitive domains in neurodegenerative diseases.

One of the strongest genetic risk factors for increased CAA scores in AD is *APOE ε4*, [15, 26, 52, 66]. This has also been studied in LBD cases, as APOE *ε*4/4 and *ε*2/4 genotypes exhibit the highest general CAA scores [25]. In the current study LBD cases with *ε*4/4 and *ε*2/4 genotypes did all exhibit CAA, although this was only a small number of cases (n = 3). Although it is worth noting that we did not include LBD cases with high AD neuropathologic change (that would be neuropathologically classified as mixed DLB/AD) to avoid a masking affect from abundant amyloid β pathology. Previous studies have discussed the masking effect of abundant amyloid pathology on an association between *APOE ε*2 and CAA severity [66], and it has been suggested that whilst APOE *ε*4 promotes vascular amyloid deposition, *ε*2 promotes progression to severe CAA and associated vasculopathic changes [22]. Interestingly *APOE ε2* has been clearly associated with CAA related haemorrhage as the frequency of the *ε2* allele is high regardless of whether significant AD related pathology was present. Aditionally in the group where significant AD pathology was present the *APOE ε2* frequency is 4 times higher than in patients with AD without haemorrhage [46]. The mechanisms behind the influence of *APOE ε2* on increased risk of cerebral haemorrhage are still yet to be elucidated, however it has been suggested that fibrinoid necrosis caused the breakage of amyloid laden vessels though its association with *APOE ε2* [41]*.*

With regards to vascular pathology, it is well known that CAA is associated with ischaemic stroke, cerebral infarction (particularly microinfarcts) in addition to haemorrhages [11, 47, 50, 60]. With increasing severity of CAA, smooth muscle and elastic elements in the vessel walls are replaced by Aβ depositions which results in fragile vessles and subsequent brain bleeds. Another consequence of Aβ in vessel walls is impaired vasoreactivity, which can lead to vessel narrowing/occlusion and hypoperfusion which may lead to ischaemic lesions in the parenchyma. When investigating the effects of severe CAA on vascular insults in the current cohort, of the 49 AD cases with severe CAA,13 were found to have infacts and microhaemorrhages, with no significant haemorrhages seen in any of the case. Out of the 4 DLB cases that exhibited CAA there were no reported vascular lesions in the neuropathological reports.

A neuroimaging study conducted by Gungor and colleagues demonstrated cerebral microbleeds (CMBs) were predominant in the occipital and frontal regions in DLB cases [23], which is in line with the finding that occipital and frontal lobes are the most frequently affected by CAA in DLB cases in the current study. Other groups have investigated the topography of CMBs across the Lewy body disease spectrum. Yamashgiro and colleagues found deep or infratentorial microbleeds were more common than lobar microbleeds (65.5% vs 34.5% respectively) in PD [65]. Although Kim and colleagues found no differences in frequency of deep or infratentorial microbleeds in DLB compared to PDD, they did demonstrate lobar microbleeds were found more frequently in DLB compared to PDD [33]. Also the occipital lobe is the brain region most commonly affected by microbleeds in DLB [53]. Interestingly, a study comparing CMBs between AD and DLB patients found there was no significant difference in the frequency of CMBs between AD and DLB, and the presence of microbleeds in DLB was not associated with amyloid deposition [16]. This suggests other mechanisms may underly the presence of microbleeds outside of general Aβ deposition, potentially the propensity of *APOE ε*2 carriers to exhibit more CAA vasculopathic changes in DLB. Taken together the results are inkeeping with the hypothesis that CAA is a common finding in DLB and may contribute to other pathological lesions and the clinical phenotype observed in these cases.

When investigating the *APOE* status in different subtypes of CAA we found *APOE ε4* carriers were more likely to have type 1 CAA in the overall cohort. This is not surprising as Thal and colleagues found the frequency of *APOE ε4* carriers in type 1 CAA is 4 times higher than in type 2 CAA in AD and controls, and type 2 CAA has a higher *APOE ε2* frequency compared to type 1 CAA [54]. Another study investigating the masking effect *APOE ε2ss* protective association with comorbid AD related pathology also ran path analysis for the presence of type 1 CAA and found no significant associations between type 1 CAA *APOE ε2*. However, this study excluded all participants with non-AD dementia, therefore the effects of *APOE ε2* in DLB warrants further investigation.

A caveat of this study is that all of the tissue used was sampled from the right hemisphere, due to the routine protocols carried out in the Newcastle Brain Tissue Resource. Several studies have observed hemispheric asymmetry in neurodegenerative pathologies that are associated with dementia [19, 34, 51]. In terms of Aβ neuroimaging Frings and colleagues demonstrate PiB retention on average was slightly higher in the right hemisphere compared to the left [19]. Whilst neuropathological studies by King and colleagues show mild asymmetry in 3/20 AD cases (with left and right hemisphere affected differently in different cases) [34]. Stefanits and colleagues demonstrate mild vulnerability of the righ hemisphere in 5/20 AD cases and 3/15 AD/DLB cases suggesting the proportion of asymmetry between cases that have AD or AD and DLB related pathologies was similar and we assume this would be similar in our cohort. We are not aware of studies that specifically investigate asymmetry of CAA, however this would be an interesting line of research.

## Conclusion

Data from the current study supports growing evidence that CAA may play an important role in the clinico-pathological phenotype of Lewy body diseases, particularly DLB. We have shown DLB cases can have more severe CAA, have an increased presence of type 1 CAA, and have a different topographical distribution of CAA, which is similar to AD, compared to PDD and PD. In conclusion data from this study suggests DLB is more aligned with AD than PDD/PD with regards to CAA severity and topographical distribution, therefore should be considered when stratifying cases for clinical trials and the design of future disease modifying therapies.

### Supplementary Information

**Additional file1.** Additional vascular lesions in the cohort.

## Data Availability

The datasets used and/or analysed during the current study available from the corresponding author on reasonable request.

## Abbreviations

Aβ: Amyloid beta
AD: Alzheimer’s disease
BA: Brodmann area
CAA: Cerebral amyloid angiopathy
CERAD: The Consortium to Establish a Registry for Alzheimer’s Disease
CMB: Cerebral microbleed
DAB: 3,3 Diaminobezidine
DLB: Dementia with Lewy bodies
LBD: Lewy body disease
NBTR: Newcastle brain tissue resource
NIA-AA: National Institute on Aging–Alzheimer’s Association
PDD: Parkinson’s disease dementia
